# Using *Staphylococcus aureus* Cas9 to Expand the Scope of Potential Gene Targets for Genome Editing in Soybean

**DOI:** 10.3390/ijms232112789

**Published:** 2022-10-24

**Authors:** Yan Zhang, Yupeng Cai, Shi Sun, Tianfu Han, Li Chen, Wensheng Hou

**Affiliations:** 1 National Center for Transgenic Research in Plants, Institute of Crop Sciences, Chinese Academy of Agricultural Sciences, Beijing 100081, China; 2 Ministry of Agriculture Key Laboratory of Soybean Biology (Beijing), Institute of Crop Sciences, Chinese Academy of Agricultural Sciences, Beijing 100081, China

**Keywords:** soybean, gene editing, CRISPR/Cas9, *Staphylococcus aureus* Cas9, PAM

## Abstract

The CRISPR (clustered regularly interspaced short palindromic repeat)/Cas (CRISPR-associated) is a revolutionary genome editing technology that has been used to achieve site-specific gene knock-out, large fragment deletion, or base editing in many plant species including soybean (*Glycine* *max*). The *Streptococcus* *pyogenes* Cas9 (SpCas9) is widely used in plants at present, although there are some reports describing the application of CRISPR/Cpf1 in soybean. Therefore, the selection range of PAM (protospacer adjacent motif) sequences for soybean is currently limited to 5′-NGG-3′ (SpCas9) or 5′-TTTN-3′ (Cpf1), which in turn limits the number of genes that can be mutated. Another Cas9 enzyme from *Staphylococcus aureus* (SaCas9) recognizes the PAM sequence 5′-NNGRRT-3′ (where R represents A or G), which can provide a wider range of potential target sequences. In this study, we developed a CRISPR/SaCas9 system and used this tool to specifically induce targeted mutations at five target sites in the *GmFT2a* (Glyma.16G150700) and *GmFT5a* (Glyma.16G044100) genes in soybean hairy roots. We demonstrated that this tool can recognize the PAM sequences 5′-AAGGGT-3′, 5′-GGGGAT-3′, 5′-TTGAAT-3′, and 5′-TAGGGT-3′ in soybean, and it achieved mutation rates ranging from 34.5% to 73.3%. Our results show that we have established a highly efficient CRISPR/SaCas9 tool that is as suitable as SpCas9 for genome editing in soybean, and it will be useful for expanding the range of target sequences for genome editing.

## 1. Introduction

The broad application of CRISPR (clustered regularly interspaced short palindromic repeats)/Cas9 (CRISPR-associated protein 9) system for genome editing in diverse organisms is a far-reaching and fundamental technological revolution in the life sciences. The diverse characteristics and continued development of CRISPR/Cas9 provide new methods to achieve site-specific gene knock-out, large fragment deletion, base editing, site-specific knock-in, target fragment replacement, and transcriptional activation and inhibition in plants [1,2,3,4].

Many previous studies have attempted to clarify how CRISPR/Cas9 system mediates RNA-guided DNA recognition and cleavage. The Cas9 protein contains two endonuclease domains, HNH and RuvC-like, which cleave the DNA strands that are complementary and non-complementary to the guide RNA, respectively [5]. Cas9-catalyzed DNA cleavage requires the recognition of a protospacer adjacent motif (PAM) located immediately downstream of the target sequence [5,6,7]. The widely used SpCas9 protein from *Streptococcus pyogenes* primarily recognizes a 5′-NGG-3′ PAM sequence [5,8]. The PAM recognition characteristics of the SpCas9 protein limit the range of possible target sequences [9]. Therefore, one feasible solution to overcome the target range limitations is to engineer Cas9 variants that recognize novel PAM sequences [9]. For example, SpCas9-VQR recognizes NGA or NGNG PAMs; SpCas9-EQR recognizes NGAG PAMs; SpCas9-VRER recognizes NGCG PAMs; SpCas9-NG recognizes NG PAMs; and xCas9 recognizes NG, GAA, and GAT PAMs [9,10,11,12]. In addition, three modified SpCas9 variants (SpCas9-NRRH, SpCas9-NRTH, and SpCas9-NRCH) have robust editing activity on non-G PAM sequences (NRNH, where R represents A or G, H represents A, C, or T) in human cells and plants [13,14]. Another SpCas9 variant named SpRY was engineered for nearly PAM less genome editing and base editing [14,15,16]. The PAM sequences recognized by the orthologous CRISPR-Cas systems are diverse. Previous studies have also reported that several CRISPR-Cas systems targeting different PAMs have been isolated from diverse microbes and engineered as effective genome editing tools in various organisms [17]. For example, the SaCas9 protein from *Staphylococcus aureus* (1053 amino acids) is smaller than SpCas9 (1368 amino acids) and is thus is easier to deliver into cells for gene editing [18]. In addition, SaCas9 recognizes a 5′-NNGRRT-3′ (where R represents A or G) PAM sequence, which is distinct from that of SpCas9 and provides additional potential target sequences [19,20]. The ScCas9 protein was isolated from *Streptococcus canis* based on its sequence similarity (89.2%) to SpCas9, and this ortholog recognizes a more relaxed PAM sequence (5′-NNG-3′) than that recognized by SpCas9 [21]. CRISPR-Cpf1 (CRISPR from *Prevotella* and *Francisella* 1) is a novel class 2 CRISPR system that recognizes a T-rich (5′-TTTN-3′) PAM sequence and cleaves DNA strands with robust genome editing activity [22,23,24].

Soybean (*Glycine max* (L.) Merr.) is an important legume crop used for food production and animal feed worldwide due to its abundant protein and oil contents. However, at present, the progress of agronomic improvement using only traditional breeding is not enough to meet the demands of a rapidly increasing human population. Thus, improving soybean breeding through innovative breeding technology is urgently needed. Although CRISPR/Cas9 systems have been widely used for soybean genome editing, SpCas9 is the main nuclease used at present [25,26]. However, there are also a few reports describing the successful application of the CRISPR/Cpf1 system in soybean [27,28]. Therefore, the range of potential PAM sequences for soybean genome editing is currently limited to 5′-NGG-3′ or 5′-TTTN-3′, which is not enough to meet the future development demand.

In this study, we developed a CRISPR/SaCas9 system and demonstrated that this tool can recognize the PAM sequences 5′-AAGGGT-3′, 5′-GGGGAT-3′, 5′-TTGAAT-3′, and 5′-TAGGGT-3′ in soybean. Our results suggest that SaCas9 can direct highly specific genome editing activity, and it will be useful for expanding the range of targets for soybean genome editing.

## 2. Results

### 2.1. Testing the Efficiency of the CRISPR/SaCas9 System for Genome Editing in Soybean

**Figure 1:**
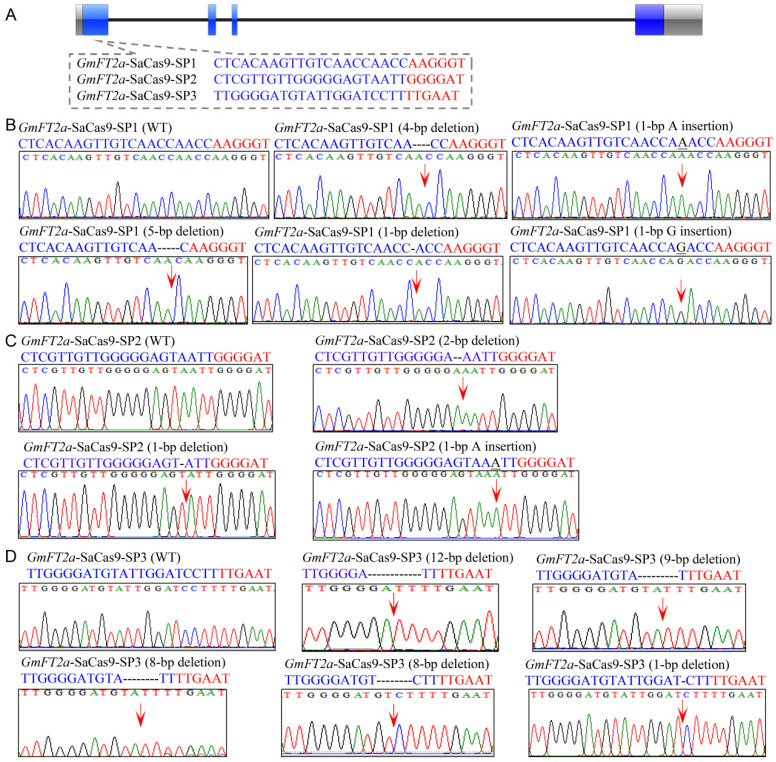
Site-specific mutations induced by the CRISPR/SaCas9 in target gene *GmFT2a*. (**A**) Gene structures of *GmFT2a* with three target sites of CRISPR/SaCas9. Blue stripe, exon; black solid line, intron; gray stripe, UTR (untranslated region). The red letters are PAM (protospacer adjacent motif) sequences. (**B**–**D**) Sequences of WT (wild type) and representative mutation types induced at target sites *GmFT2a*-SaCas9-SP1, *GmFT2a*-SaCas9-SP2 and *GmFT2a*-SaCas9-SP3 are presented, respectively. Underline, insertions. Dashes, deletions. The red arrowheads indicate the location of mutations.

**Figure 2:**
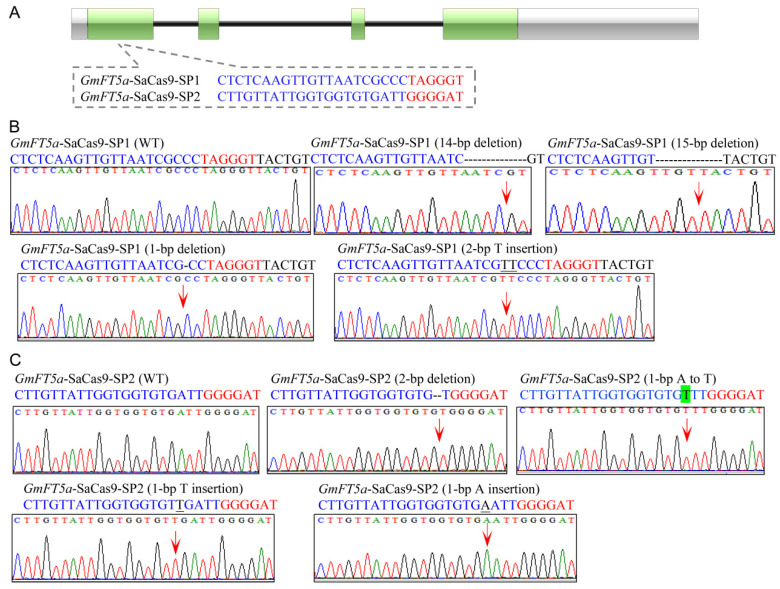
Site-specific mutations induced by the CRISPR/SaCas9 in target gene *GmFT5a*. (**A**) Gene structures of *GmFT5a* with two target sites of CRISPR/SaCas9. Green stripe, exon; black solid line, intron; gray stripe, UTR (untranslated region). The red letters are PAM (protospacer adjacent motif) sequences. (**B**,**C**) Sequences of WT (wild type) and representative mutation types induced at target sites *GmFT5a*-SaCas9-SP1 and *GmFT5a*-SaCas9-SP2 are presented, respectively. Underline, insertions. Dashes, deletions. Base substitution is indicated with the capital letter on a green background. The red arrowheads indicate the location of mutations.

In order to determine the capacity and efficiency of the CRISPR/SaCas9 system for genome editing in soybean, five sgRNAs were designed to edit different regions of two soybean genes, *GmFT2a* (Glyma.16G150700) and *GmFT5a* (Glyma.16G044100), in soybean hairy roots. Because the PAM sequence recognized by SaCas9 is 5′-NNGRRT-3′, we designed three sgRNAs (named *GmFT2a*-SaCas9-SP1, *GmFT2a*-SaCas9-SP2, and *GmFT2a*-SaCas9-SP3) that target sites located in the first exon of *GmFT2a*. The PAM sequences of these three sgRNAs are 5′-AAGGGT-3′, 5′-GGGGAT-3′, and 5′-TTGAAT-3′, respectively (Figure 1A). We also selected two sgRNAs (*GmFT5a*-SaCas9-SP1 and *GmFT5a*-SaCas9-SP2) that target sites located in the first exon of *GmFT5a*. The PAM sequences of these two sgRNAs are 5′-TAGGGT-3′ and 5′-GGGGAT-3′, respectively (Figure 2A). The five corresponding vectors targeting the aforementioned five sites were constructed and then transformed into the soybean hairy roots to induce site-specific mutations. As shown in Table 1, mutations in the *GmFT2a*-SaCas9-SP1 site were identified in 73.3% (22/30) of independently transformed transgenic hairy roots, mutations in the *GmFT2a*-SaCas9-SP2 site were identified in 34.8% (8/23) of independent transgenic hairy roots, and mutations at *GmFT2a*-SaCas9-SP3 were identified in 34.5% (10/29) of independent transgenic hairy roots. At the same time, mutations in the *GmFT5a*-SaCas9-SP1 site were identified in 42.9% (12/28) of independent transgenic hairy roots, and mutations at *GmFT5a*-SaCas9-SP2 were identified in 57.1% (16/28) of independent transgenic hairy roots.

### 2.2. Targeted Mutagenesis in GmFT2a Induced by the CRISPR/SaCas9 System

In this study, we used subcloning and DNA sequencing to detect typical types of mutations induced by CRISPR/SaCas9 in soybean hairy roots. There were five types of mutations identified at target site *GmFT2a*-SaCas9-SP1 that included a 4-bp deletion, a 5-bp deletion, a 1-bp deletion, a 1-bp A insertion, and a 1-bp G insertion (Figure 1B). Three types of mutations were identified at target site *GmFT2a*-SaCas9-SP2; a 2-bp deletion, a 1-bp deletion, and a 1-bp A insertion (Figure 1C). We also identified five types of mutations at target site *GmFT2a*-SaCas9-SP3, including a 12-bp deletion, a 9-bp deletion, an 8-bp deletion, and a 1-bp deletion (Figure 1D). These results clearly demonstrated that the CRISPR/SaCas9 system can recognize the 5′-AAGGGT-3′, 5′-GGGGAT-3′, and 5′-TTGAAT-3′ PAM sequences to induce gene site-specific knockouts in soybean.

### 2.3. Targeted Mutagenesis in GmFT5a Induced by the CRISPR/SaCas9 System

Subsequently, four types of mutations were identified at the target site *GmFT5a*-SaCas9-SP1. These were a 14-bp deletion, a 15-bp deletion, a 1-bp deletion, and a 2-bp T insertion (Figure 2B). Moreover, four types of mutations were identified at the target site *GmFT5a*-SaCas9-SP2, including a 2-bp deletion, a 1-bp T insertion, a 1-bp A insertion, and a 1-bp transversion (A changed to T) (Figure 2C). These results demonstrated that the CRISPR/SaCas9 system can recognize the 5′-TAGGGT-3′ and 5′-GGGGAT-3′ PAM sequences to induce gene site-specific knockouts in soybean.

## 3. Discussion

### 3.1. SaCas9 Has Comparable Genome Editing Efficiency to SpCas9 in Soybean

The successful application of a new genome editing tool requires that it be efficient at inducing targeted mutations. In 2015, CRISPR/SpCas9 was first used to knock out target genes in soybean hairy roots. Targeted DNA mutations were detected in 95% of 88 hairy-root transgenic events analyzed [29]. To select a more suitable promoter for the CRISPR/SpCas9 system in soybean, the soybean *U6-10* and *Arabidopsis U6-26* promoters were compared. Mutation efficiencies ranged from 3.2 to 9.7% using the *Arabidopsis U6-26* promoter and 14.7–20.2% with the soybean *U6-10* promoter [30]. Similarly, mutation efficiencies of 43.4–48.1% were achieved with CRISPR/SpCas9 using the soybean *U6-16g-1* promoter in two editing targets, *GmPDS11* and *GmPDS18* [31]. In a previous study, we also demonstrated that CRISPR/SpCas9 can be successfully applied to generate mutations in the desired target genes (*bar* transgene, *GmFEI1/2*, and *GmSHR*) in soybean hairy roots with mutagenesis efficiencies of 10.0–93.3% [32]. In this study, we developed a CRISPR/SaCas9 system and employed this tool to specifically induce targeted mutations in five target sites of two soybean genes, *GmFT2a* and *GmFT5a*, and achieved mutation frequencies ranging from 34.5% to 73.3%. These results indicated that the efficiency of the SaCas9 enzyme is comparable to that of SpCas9 in soybean; that is to say, SaCas9 is equally suitable for genome editing in soybean.

### 3.2. CRISPR/SaCas9 Can Be Used to Expand the Targeting Scope for Soybean Genome Editing

In recent years, the use of various CRISPR technologies in soybean has been widely reported. CRISPR/SpCas9 has been successfully used to achieve the site-specific knockout of target genes in both soybean hairy roots and regenerated plants for improving important agronomic characteristics [29,32,33]. For example, the CRISPR/SpCas9 tool was used to broaden the latitudinal adaptability of soybean by regulating flowering time. Plants carrying the *ft2a*, *ft2b*, and *ft5a* mutations generated by CRISPR/SpCas9 exhibit late flowering phenotypes [33,34,35]. Early flowering soybean mutants have also been generated with CRISPR/Cas9 technology by knocking out *E1* [36]. The soybean quadruple *lcls* mutants generated by CRISPR/SpCas9 exhibit an extreme short-period circadian rhythm and a late flowering phenotype [37]. The *gmprr37* mutants generated by CRISPR/SpCas9 show an early flowering phenotype under long-day conditions [38]. CRISPR/SpCas9 has also been used to optimize soybean plant architecture (plant height, internode length, and the number of nodes and branches) by knocking out four *SQUAMOSA PROMOTER BINDING PROTEIN-LIKE 9* (*SPL9*) homologues and four soybean *LATE ELONGATED HYPOCOTYL* (*LHY*) genes [39,40]. CRISPR/SpCas9 could also be used to improve the quality of soybean seeds, such as by regulating the oleic acid content and the taste of soybean oil and protein products [41,42]. However, SpCas9 can only recognize the 5′-NGG-3′ PAM sequences, which means that in some cases, it cannot meet the needs of target gene selection.

In addition to single-gene knock-out, the CRISPR/SpCas9-mediated deletion of large genomic fragments and target base editing have also been successfully applied to soybean to improve agronomic traits [43,44]. The deletion of large DNA fragments can be used to study the function of specific sequence elements in genes. In addition, base editing is routinely used to regulate gene function by targeting specific single nucleotides. Therefore, compared to gene knock-out, an expanded range of PAM sites is required for the deletion of specific DNA sequence elements or for base editing. In this study, we developed a CRISPR/SaCas9 system and demonstrated that this tool can recognize the PAM sequences 5′-AAGGGT-3′, 5′-GGGGAT-3′, 5′-TTGAAT-3′, and 5′-TAGGGT-3′ in soybean genomic DNA. Moreover, the CRISPR/SaCas9 system will be more accurate in creating mutations, because SaCas9 recognizes a longer PAM motif than SpCas9. Our results suggest that SaCas9 can direct highly specific genome editing activity, and it will therefore be useful for expanding the scope of potential target genes for genome editing in soybean.

## 4. Materials and Methods

### 4.1. SgRNA Design and Construction of the CRISPR/SaCas9 Vectors

The genome sequences and detailed information of the soybean genes *GmFT2a* and *GmFT5a* were downloaded from the Phytozome website (www.phytozome.net, accessed on 11 March 2021). Because the PAM sequence recognized by SaCas9 is 5′-NNGRRT-3′ (where R represents A or G), we designed three sgRNAs (named *GmFT2a*-SaCas9-SP1, *GmFT2a*-SaCas9-SP2, and *GmFT2a*-SaCas9-SP3) to target three sites located in the first exon of *GmFT2a*. The PAM sequences of these three sgRNAs are 5′-AAGGGT-3′, 5′-GGGGAT-3′ and 5′-TTGAAT-3′, respectively. We also designed two sgRNAs (*GmFT5a*-SaCas9-SP1 and *GmFT5a*-SaCas9-SP2) that target sites located in the first exon of *GmFT5a*. The PAM sequences of these two sgRNAs are 5′-TAGGGT-3′ and 5′-GGGGAT-3′, respectively. The specificity of these five target sequences were identified by sequence comparison with the soybean genome database in the NCBI website (www.ncbi.nlm.nih.gov, accessed on 11 March 2021). For each sgRNA, a pair of DNA oligos were synthesized by Tsingke Biotechnology (Beijing, China) and annealed to generate dimers using PCR amplification. These dimers were subsequently ligated upstream of the sgRNA scaffolds in the plasmid vector for the simultaneous expression of SaCas9 and sgRNA. The sgRNA expression cassette was driven by the *Arabidopsis U6* promoter (Supplementary Data S1). The SaCas9 gene sequence with two NLS (nuclear localization signal) sequences was codon-optimized for dicotyledons (Supplementary Data S2) and placed downstream of the CaMV 2× 35S promoter. The *GFP* gene driven by the CaMV 35S promoter was used for the rapid visual screening of transgenic hairy roots.

### 4.2. Hairy Root Transformation Using Agrobacterium rhizogenes K599

The corresponding CRISPR/SaCas9 vectors were introduced into *Agrobacterium rhizogenes* K599 via electroporation. The soybean cultivar ‘Jack’ was used for hairy root transformation in the present study according to the protocol previously described [32,45]. After cultivation for 15 days, each individual hairy root that had grown to a length of 5 to 6 cm was numbered and examined using a stereoscopic fluorescence microscope (Nikon SMZ1500, Nikon, Tokyo, Japan). The hairy roots harboring the desired CRISPR/SaCas9 vectors gave GFP fluorescence signals (Supplementary Figure S1) and were used for further analysis.

### 4.3. Determination of the Site-Specific Mutations Induced by the CRISPR/SaCas9 in Target Genes

Each transgenic hairy root is an independent transformation event. The genomic DNA of each transgenic hairy root was extracted using the CTAB method and subsequently used as templates for PCR amplification with specific primers followed by DNA sequencing of the amplicons. To detect mutations in the *GmFT2a* gene, the primers *GmFT2a*-SaCas9-F (5′-AAGCAAACGAGTATATAAGAAAGCA-3′) and *GmFT2a*-SaCas9-R (5′-TGGATGGTCAAAAACAATAACGTC-3′) were designed to amplify a 585 bp amplicon containing the three target sequences *GmFT2a*-SaCas9-SP1, *GmFT2a*-SaCas9-SP2, and *GmFT2a*-SaCas9-SP3. To detect mutations in the *GmFT5a* gene, the primers *GmFT5a*-SaCas9-F (5′-GCAGATGCTAAGGTGGAAAAATA-3′) and *GmFT5a*-SaCas9-R (5′-TGCATCCACCATAACCTGAGAT-3′) were designed to amplify a 462 bp amplicon containing the two target sequences *GmFT5a*-SaCas9-SP1 and *GmFT5a*-SaCas9-SP2. The heterozygous mutations showed overlapping peaks from the target sites to the end, while the wild-type and homozygous mutations had no overlapping peaks at the targeted sites [26]. The mutation rates were calculated using the following formula: mutation rate = number of hairy roots with mutations/number of hairy roots examined × 100%. In order to identify the specific types of mutations, the PCR products were further characterized by subcloning, DNA sequencing and alignment with the reference sequence.

## Figures and Tables

**Table 1:** Summary of CRISPR/SaCas9 genome editing assays in the present study.

Target Gene	Target Site	No. of Hairy Roots Examined	No. of Hairy Roots with Mutations	Mutation Rates
*GmFT2a*	*GmFT2a*-SaCas9-SP1	30	22	73.3%
*GmFT2a*	*GmFT2a*-SaCas9-SP2	23	8	34.8%
*GmFT2a*	*GmFT2a*-SaCas9-SP3	29	10	34.5%
*GmFT5a*	*GmFT5a*-SaCas9-SP1	28	12	42.9%
*GmFT5a*	*GmFT5a*-SaCas9-SP2	28	16	57.1%

## Supplementary Materials

The following supporting information can be downloaded at: https://www.mdpi.com/article/10.3390/ijms232112789/s1.

## References

[1] Shan Q., Wang Y., Li J., Zhang Y., Chen K., Liang Z., Zhang K., Liu J., Xi J.J., Qiu J.-L. (2013). Targeted genome modification of crop plants using a CRISPR-Cas system. Nat. Biotechnol..

[2] Li J., Meng X., Zong Y., Chen K., Zhang H., Liu J., Gao C. (2016). Gene replacements and insertions in rice by intron targeting using CRISPR-Cas9. Nat. Plants.

[3] Kiani S., Chavez A., Tuttle M., Hall R.N., Chari R.S., Ter-Ovanesyan D., Qian J., Pruitt B., Beal J.S., Vora S.D. (2015). Cas9 gRNA engineering for genome editing, activation and repression. Nat. Methods.

[4] Rodríguez-Leal D., Lemmon Z.H., Man J., Bartlett M.E., Lippman Z.B. (2017). Engineering Quantitative Trait Variation for Crop Improvement by Genome Editing. Cell.

[5] Jinek M., Chylinski K., Fonfara I., Hauer M., Doudna J.A., Charpentier E. (2012). A Programmable dual-RNA-guided DNA endonuclease in adaptive bacterial immunity. Science.

[6] Mali P., Yang L., Esvelt K.M., Aach J., Guell M., DiCarlo J.E., Norville J.E., Church G.M. (2013). RNA-Guided Human Genome Engineering via Cas9. Science.

[7] Mojica F.J.M., Díez-Villaseñor C., García-Martínez J., Almendros C. (2009). Short motif sequences determine the targets of the prokaryotic CRISPR defence system. Microbiology.

[8] Sternberg S.H., Redding S., Jinek M., Greene E.C., Doudna J.A. (2014). DNA interrogation by the CRISPR RNA-guided endonuclease Cas9. Nature.

[9] Kleinstiver B., Prew M.S., Tsai S.Q., Topkar V.V., Nguyen N.T., Zheng Z., Gonzales A.P.W., Li Z., Peterson R.T., Yeh J.-R.J. (2015). Engineered CRISPR-Cas9 nucleases with altered PAM specificities. Nature.

[10] Yamamoto A., Ishida T., Yoshimura M., Kimura Y., Sawa S. (2019). Developing Heritable Mutations in Arabidopsis thaliana Using a Modified CRISPR/Cas9 Toolkit Comprising PAM-Altered Cas9 Variants and gRNAs. Plant Cell Physiol..

[11] Hu J.H., Miller S.M., Geurts M.H., Tang W., Chen L., Sun N., Zeina C.M., Gao X., Rees H.A., Lin Z. (2018). Evolved Cas9 variants with broad PAM compatibility and high DNA specificity. Nature.

[12] Zeng D., Liu T., Tan J., Zhang Y., Zheng Z., Wang B., Zhou D., Xie X., Guo M., Liu Y.-G. (2020). PhieCBEs: Plant High-Efficiency Cytidine Base Editors with Expanded Target Range. Mol. Plant.

[13] Miller S.M., Wang T., Randolph P.B., Arbab M., Shen M.W., Huang T.P., Matuszek Z., Newby G.A., Rees H.A., Liu D.R. (2020). Continuous evolution of SpCas9 variants compatible with non-G PAMs. Nat. Biotechnol..

[14] Zhang C., Wang Y., Wang F., Zhao S., Song J., Feng F., Zhao J., Yang J. (2020). Expanding base editing scope to near-PAMless with engineered CRISPR/Cas9 variants in plants. Mol. Plant.

[15] Walton R.T., Christie K.A., Whittaker M.N., Kleinstiver B.P. (2020). Unconstrained genome targeting with near-PAMless engineered CRISPR-Cas9 variants. Science.

[16] Ren J., Meng X., Hu F., Liu Q., Cao Y., Li H., Yan C., Li J., Wang K., Yu H. (2021). Expanding the scope of genome editing with SpG and SpRY variants in rice. Sci. China Life Sci..

[17] Murovec J., Pirc Ž., Yang B. (2017). New variants of CRISPR RNA-guided genome editing enzymes. Plant Biotechnol. J..

[18] Ran F.A., Cong L., Yan W.X., Scott D.A., Gootenberg J., Kriz A.J., Zetsche B., Shalem O., Wu X., Makarova K.S. (2015). In vivo genome editing using *Staphylococcus aureus* Cas9. Nature.

[19] Nishimasu H., Cong L., Yan W.X., Ran F.A., Zetsche B., Li Y., Kurabayashi A., Ishitani R., Zhang F., Nureki O. (2015). Crystal Structure of *Staphylococcus aureus* Cas9. Cell.

[20] Kleinstiver B.P., Prew M.S., Tsai S.Q., Nguyen N.T., Topkar V.V., Zheng Z., Joung J.K. (2015). Broadening the targeting range of *Staphylococcus aureus* CRISPR-Cas9 by modifying PAM recognition. Nat. Biotechnol..

[21] Chatterjee P., Jakimo N., Jacobson J.M. (2018). Minimal PAM specificity of a highly similar SpCas9 ortholog. Sci. Adv..

[22] Zetsche B., Gootenberg J.S., Abudayyeh O.O., Slaymaker I.M., Makarova K.S., Essletzbichler P., Volz S.E., Joung J., van der Oost J., Regev A. (2015). Cpf1 Is a Single RNA-Guided Endonuclease of a Class 2 CRISPR-Cas System. Cell.

[23] Fagerlund R.D., Staals R.H.J., Fineran P.C. (2015). The Cpf1 CRISPR-Cas protein expands genome-editing tools. Genome Biol..

[24] Wang M., Mao Y., Lu Y., Tao X., Zhu J.-K. (2017). Multiplex Gene Editing in Rice Using the CRISPR-Cpf1 System. Mol. Plant.

[25] Chilcoat D., Liu Z.-B., Sander J., Weeks D.P., Yang B. (2017). Chapter Two—Use of CRISPR/Cas9 for crop improvement in maize and soybean. Progress in Molecular Biology and Translational Science.

[26] Chen L., Cai Y., Hou W., Islam M.T., Molla K.A. (2021). Generation of knockout and fragment deletion mutants in soybean by CRISPR-Cas9. CRISPR-Cas Methods.

[27] Duan K., Cheng Y., Ji J., Wang C., Wei Y., Wang Y. (2021). Large chromosomal segment deletions by CRISPR/LbCpf1-mediated multiplex gene editing in soybean. J. Integr. Plant Biol..

[28] Kim H., Kim S.-T., Ryu J., Kang B.-C., Kim J.-S., Kim S.-G. (2017). CRISPR/Cpf1-mediated DNA-free plant genome editing. Nat. Commun..

[29] Jacobs T.B., Lafayette P.R., Schmitz R.J., A Parrott W. (2015). Targeted genome modifications in soybean with CRISPR/Cas9. BMC Biotechnol..

[30] Sun X., Hu Z., Chen R., Jiang Q., Song G., Zhang H., Xi Y. (2015). Targeted mutagenesis in soybean using the CRISPR-Cas9 system. Sci. Rep..

[31] Du H., Zeng X., Zhao M., Cui X., Wang Q., Yang H., Cheng H., Yu D. (2016). Efficient targeted mutagenesis in soybean by TALENs and CRISPR/Cas9. J. Biotechnol..

[32] Cai Y., Chen L., Liu X., Sun S., Wu C., Jiang B., Han T., Hou W. (2015). CRISPR/Cas9-Mediated Genome Editing in Soybean Hairy Roots. PLoS ONE.

[33] Cai Y., Chen L., Liu X., Guo C., Sun S., Wu C., Jiang B., Han T., Hou W. (2018). CRISPR/Cas9-mediated targeted mutagenesis of *GmFT2a* delays flowering time in soya bean. Plant Biotechnol. J..

[34] Chen L., Cai Y., Qu M., Wang L., Sun H., Jiang B., Wu T., Liu L., Sun S., Wu C. (2020). Soybean adaption to high-latitude regions is associated with natural variations of GmFT2b, an ortholog of FLOWERING LOCUS T. Plant Cell Environ..

[35] Cai Y., Wang L., Chen L., Wu T., Liu L., Sun S., Wu C., Yao W., Jiang B., Yuan S. (2020). Mutagenesis of GmFT2a and GmFT5a mediated by CRISPR/Cas9 contributes for expanding the regional adapt-ability of soybean. Plant Biotechnol. J..

[36] Han J., Guo B., Guo Y., Zhang B., Wang X., Qiu L.-J. (2019). Creation of Early Flowering Germplasm of Soybean by CRISPR/Cas9 Technology. Front. Plant Sci..

[37] Wang Y.U., Yuan L.I., Su T., Wang Q., Gao Y.A., Zhang S., Jia Q., Yu G., Fu Y., Cheng Q. (2020). Light- and temperature-entrainable circadian clock in soybean development. Plant Cell Environ..

[38] Wang L., Sun S., Wu T., Liu L., Sun X., Cai Y., Li J., Jia H., Yuan S., Chen L. (2020). Natural variation and CRISPR/Cas9-mediated mutation in *GmPRR37* affect photoperiodic flowering and contribute to regional adaptation of soybean. Plant Biotechnol. J..

[39] Bao A., Chen H., Chen L., Chen S., Hao Q., Guo W., Qiu D., Shan Z., Yang Z., Yuan S. (2019). CRISPR/Cas9-mediated targeted mutagenesis of GmSPL9 genes alters plant architecture in soybean. BMC Plant Biol..

[40] Cheng Q., Dong L., Su T., Li T., Gan Z., Nan H., Lu S., Fang C., Kong L., Li H. (2019). CRISPR/Cas9-mediated targeted mutagenesis of GmLHY genes alters plant height and internode length in soybean. BMC Plant Biol..

[41] Al Amin N., Ahmad N., Wu N., Pu X., Ma T., Du Y., Bo X., Wang N., Sharif R., Wang P. (2019). CRISPR-Cas9 mediated targeted disruption of FAD2–2 microsomal omega-6 desaturase in soybean (*Glycine max.* L.). BMC Biotechnol..

[42] Wang J., Kuang H., Zhang Z., Yang Y., Yan L., Zhang M., Song S., Guan Y. (2020). Generation of seed lipoxygenase-free soybean using CRISPR-Cas9. Crop J..

[43] Cai Y., Chen L., Zhang Y., Yuan S., Su Q., Sun S., Wu C., Yao W., Han T., Hou W. (2020). Target base editing in soybean using a modified CRISPR/Cas9 system. Plant Biotechnol. J..

[44] Cai Y., Chen L., Sun S., Wu C., Yao W., Jiang B., Han T., Hou W. (2018). CRISPR/Cas9-Mediated Deletion of Large Genomic Fragments in Soybean. Int. J. Mol. Sci..

[45] Chen L., Cai Y., Liu X., Guo C., Sun S., Wu C., Jiang B., Han T., Hou W. (2018). Soybean hairy roots produced in vitro by Agrobacterium rhizogenes-mediated transformation. Crop J..

