# Supplementary material for: Using Staphylococcus aureus Cas9 to Expand the Scope of Potential Gene Targets for Genome Editing in Soybean
Source: Int J Mol Sci. 2022 Oct 24;23(21):12789. doi: 10.3390/ijms232112789 (PMC9658631; doi:10.3390/ijms232112789)
Supplement: Supplementary file 1 [file ijms-23-12789-s001.zip › ijms-1927570-supplementary.pdf]

Supplementary data S1. The sequence of the expression cassette of sgRNA.

TTCGTTGAACAACGGAAACTCGACTTGCCTTCCGCACAATACATCATTTCTTCTTAGCTTTTTTTCTTCTTCT
TCGTTCATACAGTTTTTTTTTGTTTATCAGCTTACATTTTCTTGAACCGTAGCTTTCGTTTTCTTCTTTTTAAC
TTTCCATTCGGAGTTTTTGTATCTTGTTTCATAGTTTGTCCCAGGATTAGAATGATTAGGCATCGAACCTTCA
AGAATTTGATTGAATAAAACATCTTCATTCTTAAGATATGAAGATAATCTTCAAAAGGCCCCTGGGAATCTG
AAAGAAGAGAAGCAGGCCCATTTATATGGGAAAGAACAATAGTATTTCTTATATAGGCCCATTTAAGTTGAA
AACAATCTTCAAAAGTCCCACATCGCTTAGATAAGAAAACGAAGCTGAGTTTATATACAGCTAGAGTCGAA
GTAGTGATTGNNNNNNNNNNNNNNNNNNNNGTTTTAGTACTCTGGAAACAGAATCTACTAAAACAAGGCA
AAATGCCGTGTTTATCTCGTCAACTTGTTGGCGAGATTTTTTT

Blue letters indicate the *Arabidopsis U6* promoter. Red letters indicate the sgRNA scaffold. The capital letters on a yellow background will be replaced by target sequences.

Supplementary data S2. The sequences of SaCas9 and NLS used in this study.

ATGGCCCCAAAGAAGAAGAGAAAGGTCGGTATTCATGGAGTCCCAGCAGCCAAGAGAAACTACATCCTGG
GACTGGACATCGGAATCACCAGTGTGGGATACGGAATCATCGACTACGAGACAAGAGACGTGATCGATGC
CGGAGTGAGACTGTTCAAAGAGGCCAACGTGGAAAACAACGAGGGAAGGAGAAGTAAGAGAGGAGCCA
GAAGGCTGAAGAGAAGAAGGAGACATAGAATCCAGAGAGTGAAGAAGCTGCTGTTCGACTACAACCTGC
TGACCGACCATAGTGAGCTGAGTGGAATCAACCCATACGAGGCCAGAGTGAAGGGACTGAGTCAGAAGC
TGAGTGAGGAAGAGTTCTCTGCCGCCCTGCTGCATCTGGCCAAGAGAAGAGGAGTGCATAACGTGAACGA
GGTGGAAGAGGACACCGGAAACGAGCTGTCCACCAAAGAGCAGATCAGTAGAAACAGTAAGGCCCTGGA
AGAGAAATACGTGGCCGAACTGCAGCTGGAAAGACTGAAGAAAGACGGAGAAGTGAGAGGAAGTATCA
ACAGATTCAAGACCAGTGACTACGTGAAAGAAGCCAAACAGCTGCTGAAGGTGCAGAAGGCCTACCATC
AGCTGGACCAGAGTTTCATCGACACCTACATCGACCTGCTGGAAACCAGAAGAACCTACTATGAGGGACC
TGGAGAGGGAAGTCCATTCGGATGGAAGGACATCAAAGAATGGTACGAGATGCTGATGGGACATTGTACC
TACTTCCCAGAGGAACTGAGAAGTGTGAAGTACGCCTACAACGCCGACCTGTACAACGCCCTGAACGACC
TGAACAATCTCGTGATCACCAGGGACGAGAACGAGAAGCTGGAATATTACGAGAAGTTCCAGATCATCGA
GAACGTGTTCAAGCAGAAGAAGAAGCCAACCCTGAAGCAGATCGCCAAAGAAATCCTCGTGAACGAAGA
GGATATTAAGGGATACAGAGTGACCAGTACCGGAAAGCCAGAGTTCACCAACCTGAAGGTGTACCATGAC
ATCAAGGACATTACCGCCAGAAAAGAGATTATTGAGAACGCCGAGCTGCTGGATCAGATTGCCAAGATCCT
GACCATCTACCAGAGTAGTGAGGACATCCAGGAAGAACTGACCAATCTGAACTCCGAGCTGACCCAGGAA
GAGATCGAGCAGATCTCTAATCTGAAGGGATATACCGGAACCCATAACCTGAGTCTGAAGGCCATCAACCT
GATCCTGGACGAGCTGTGGCATACCAACGACAACCAGATCGCTATCTTCAACAGACTGAAGCTGGTGCCA
AAGAAGGTGGACCTGTCCCAGCAGAAAGAGATCCCAACCACCCTGGTGGACGACTTCATCCTGAGTCCAG
TCGTGAAGAGAAGTTTCATCCAGAGTATCAAAGTGATCAACGCCATCATCAAGAAGTACGGACTGCCAAA
CGACATCATTATCGAGCTGGCCAGAGAGAAGAACTCCAAGGACGCCCAGAAAATGATCAACGAGATGCAG
AAGAGAAACAGACAGACCAACGAGAGAATCGAGGAAATCATCAGAACCACCGGAAAAGAGAACGCCAA
GTACCTGATCGAGAAGATCAAGCTGCATGACATGCAGGAAGGAAAGTGTCTGTACAGTCTGGAAGCCATC
CCTCTGGAAGATCTGCTGAACAACCCATTCAACTATGAGGTGGACCATATCATCCCAAGAAGTGTGTCCTT
CGACAACAGTTTCAACAACAAGGTGCTCGTGAAGCAGGAAGAAAACAGTAAGAAGGGAAACAGAACCC

CATTCCAGTACCTGAGTAGTAGTGACAGTAAGATCAGTTACGAAACCTTCAAGAAGCATATCCTGAATCTG
GCCAAGGGAAAGGGAAGAATCAGTAAGACCAAGAAAGAGTATCTGCTGGAAGAAAGAGACATCAACAG
GTTCTCCGTGCAGAAAGACTTCATCAACAGAAACCTGGTGGATACCAGATACGCCACCAGAGGACTGATG
AACCTGCTGAGAAGTTACTTCAGAGTGAACAACCTGGACGTGAAAGTGAAGTCCATCAATGGAGGATTCA
CCAGTTTTCTGAGAAGAAAGTGGAAGTTTAAGAAAGAGAGAAACAAGGGTTACAAGCATCATGCCGAGG
ACGCCCTGATCATTGCCAACGCCGATTTCATCTTCAAAGAGTGGAAGAAACTGGACAAGGCCAAAAAAGT
GATGGAAAACCAGATGTTCGAGGAAAAGCAGGCCGAGAGTATGCCAGAGATCGAAACCGAGCAGGAGTA
CAAAGAGATCTTCATCACCCCACATCAGATCAAGCATATTAAGGACTTCAAGGACTACAAGTACAGTCATA
GAGTGGACAAGAAGCCTAATAGAGAGCTGATTAACGACACCCTGTACTCCACCAGAAAGGACGACAAGG
GAAACACCCTGATCGTGAACAATCTGAACGGACTGTACGACAAGGACAATGACAAGCTGAAAAAGCTGAT
CAACAAGAGTCCAGAAAAGCTGCTGATGTACCATCATGACCCACAGACCTACCAGAAACTGAAGCTGATT
ATGGAACAGTACGGAGACGAGAAGAATCCACTGTACAAGTACTACGAGGAAACCGGTAACTACCTGACCA
AGTACTCCAAAAAGGACAACGGACCAGTGATCAAGAAGATTAAGTATTACGGAAACAAACTGAACGCCCA
TCTGGACATCACCGACGACTACCCAAACAGTAGAAACAAGGTCGTGAAGCTGTCCCTGAAGCCATACAGA
TTCGACGTGTACCTGGACAATGGAGTGTACAAGTTCGTGACCGTGAAGAATCTGGATGTGATCAAAAAAG
AAAACTACTACGAAGTGAATAGTAAGTGTTATGAGGAAGCTAAGAAGCTGAAGAAGATCAGTAACCAGGC
CGAGTTTATCGCCTCCTTCTACAACAACGATCTGATCAAGATCAACGGAGAGCTGTATAGAGTGATCGGAG
TGAACAACGACCTGCTGAACAGAATCGAAGTGAACATGATCGACATCACCTACAGAGAGTACCTGGAAAA
CATGAACGACAAGAGGCCACCAAGGATCATTAAGACAATCGCCTCCAAGACCCAGAGTATTAAGAAGTAC
AGTACAGACATTCTGGGAAACCTGTATGAAGTGAAATCTAAGAAGCATCCTCAGATCATCAAAAAGGGAA
AAAGGCCTGCTGCCACTAAAAAGGCCGGACAGGCAAAAAAGAAAAAGTAA

The sequence of SV40 NLS is indicated with the capital letter on a yellow background, and the sequence of nucleoplasmin NLS is indicated with the capital letter on a green background.

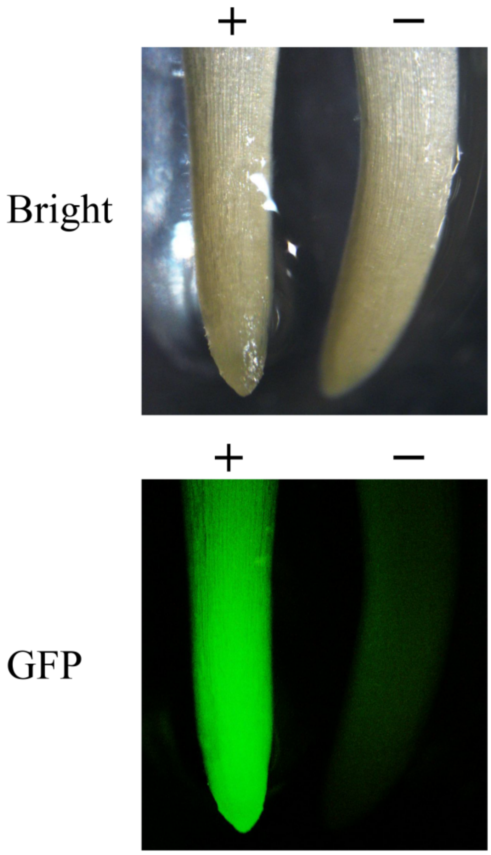

Supplementary Figure S1. Characterization of transgenic hairy roots with a GFP fluorescence labeling. +, transgenic hairy root. -, negative control.
